# Clinical Benefit of Pembrolizumab in Advanced Urothelial Cancer Patients in Real-Life Setting: An Efficacy and Safety Monocentric Study

**DOI:** 10.3390/curroncol29020080

**Published:** 2022-02-10

**Authors:** Elodie Dang, Alexandre Vallée, Coralie Lepage-Seydoux, Karine Sejean, Brigitte Bonan, Christine Abraham, Philippe Beuzeboc, Raffaele Ratta

**Affiliations:** 1 Pharmacy Department, Foch Hospital, 92150 Suresnes, France; elodie.dang@gmail.com (E.D.); c.lepage@hopital-foch.com (C.L.-S.); k.sejean@hopital-foch.com (K.S.); b.bonan@hopital-foch.com (B.B.); 2 Clinical Research and Innovation Department, Foch Hospital, 92150 Suresnes, France; al.vallee@hopital-foch.com; 3 Medical Oncology Department, Foch Hospital, 92150 Suresnes, France; c.abraham@hopital-foch.com (C.A.); p.beuzeboc@hopital-foch.com (P.B.)

**Keywords:** immune checkpoint inhibitors, immunotherapy, pembrolizumab, programmed cell death 1 receptor, urinary bladder neoplasms

## Abstract

Background: Pembrolizumab is approved for patients with metastatic urothelial carcinoma (UC) who progressed under platinum therapy. The aim of this study was to assess the efficacy and safety of pembrolizumab in a cohort of real-life UC patients. Methods: This retrospective, observational study included advanced UC patients treated with pembrolizumab in a single institution in France. The co-primary endpoints were overall survival (OS) and progression-free survival (PFS) at 6 months. Secondary endpoints were objective response rate (ORR), duration of response (DOR), disease control rate (DCR) and safety. Results: 78 patients were included in the study. The median OS was 7.3 months (3.8–12.2). The estimated OS rate at 6 months was 61.5% (50.5–72.6). The median PFS was 3.1 months (1.4–7.2). The estimated PFS rate at 6 months was 42.3% (31.1–53.5). The best ORR was 35.9%. The mean DOR was 95.5 days. The DCR was 30.8%. The most common treatment-related adverse events (AEs) of any grade were fatigue (46.2%), diarrhea (11.5%), pruritus (10.3%) and nausea (9.0%). There were no grade 3 AEs that occurred with an incidence of 5% or more. Conclusion: Our results confirmed those of randomized clinical trials concerning the treatment with pembrolizumab in patients with advanced UC that progressed after platinum-based chemotherapy.

## 1. Introduction

Metastatic urothelial carcinoma (UC) is a highly lethal disease with a poor prognosis and a 5-year overall survival (OS) of less than 5% [1]. Urothelial carcinoma of the bladder (UCB) is the most common urinary tract cancer. The upper tract urothelial carcinoma (UTUC), which involves the renal pelvis and ureter, is less common than UCB but it is usually more invasive at diagnosis.

Before the advent of first-line platinum-based chemotherapy strategies, locally advanced or metastatic UC were associated with a median survival of 3 to 6 months.

The development in the last years of immune checkpoint inhibitors (ICIs), blocking the interaction of Programmed Death 1 (PD-1) and Cytotoxic T-Lymphocyte Antigen 4 (CTLA-4) with their ligands, has revolutionized the treatment of several solid tumors, including UC [2,3]. Intravesical instillation of Bacillus of Calmette-Guérin (BCG) in high-risk, non-muscle invasive disease has shown that UC could be a suitable candidate for modern immunotherapy [4]; moreover, UC has a high immunogenicity, an element which supports the application of ICIs in the advanced or metastatic setting [5].

The advent of immunotherapy has changed the treatment paradigm and the prognosis of UC patients. A number of immune checkpoint inhibitors (ICIs) have been approved as first-line therapy in cisplatin-ineligible patients or as second-line therapy after platinum-based treatments [6,7], but also in the adjuvant setting [8].

Pembrolizumab is a humanized IgG4 monoclonal antibody against PD-1, blocking its engagement with its ligands (PD-L1 and PD-L2). It has been recently approved by the European Medicines Agency as monotherapy for the treatment of locally advanced or metastatic UC in adults who have received prior platinum-based chemotherapy and for treatment of locally advanced or metastatic UC in adults who are not eligible for cisplatin-containing chemotherapy and whose tumors express PD-L1 with a combined positive score (CPS) ≥ 10.

Pembrolizumab has shown a robust antitumor activity and a good safety profile in UC in the phase 1b Keynote-012 study [9] and in the phase 2 Keynote-052 study [10]. The results of these two trials led to the randomized, phase 3 Keynote-045 trial [11], an open label study in which 542 selected patients who progressed under platinum therapy were randomized to receive pembrolizumab at a dose of 200 mg every 3 weeks or chemotherapy. In the Keynote-045 trial, the median OS (mOS) was longer in the pembrolizumab arm compared to the chemotherapy group (10.3 months vs. 7.4 months, *p* = 0.002), whereas the median progression-free survival (mPFS) was not superior for pembrolizumab in comparison to chemotherapy. However, the objective response rate (ORR) for the pembrolizumab group was higher than the chemotherapy group (21.1% vs. 11.4%, *p* = 0.001). After at least 5 years of follow-up, pembrolizumab continued to show improved OS, ORR and duration of response (DOR) [12].

Here, we provided complementary information to previous randomized controlled trials (RCTs) in a real-world study, whose aim was to assess the efficacy and safety of pembrolizumab in a cohort of real-life patients with UC treated in a single hospital in France.

## 2. Materials and Methods

### 2.1. Study Design

This retrospective, observational, monocentric study included UC patients treated at Foch hospital in Suresnes, France. We provided to all patients written information about the study’s objectives and the nature of the information that we collected. The study was designed by the oncology department. Data were collected by the pharmacy and oncology department.

The study was approved by Foch IRB: IRB00012437 (approval number: 21-02-01) on 26 February 2021, a non-opposed consent was obtained for all participants.

The study was conducted in accordance with the protocol and its amendments, with Good Clinical Practice guidelines and with the provisions of the Declaration of Helsinki.

### 2.2. Patients

We collected data from patients aged 18 years or older, who presented a metastatic UC of the ureter, bladder or urethra, treated with pembrolizumab, who had a disease progression after at least one platinum-based chemotherapy used in the neoadjuvant, adjuvant or metastatic setting. Patients were ineligible if they received pembrolizumab as first line treatment before any other chemotherapy. Treatment efficacy was assessed by physician’s clinical evaluation and computerized-tomography scans (CT-scans) on a regular basis. ORR was assessed by radiological exams locally performed and using the Response Evaluation Criteria In Solid Tumor (RECIST) version 1.1. Patients without a follow-up scan after beginning of pembrolizumab were considered as not evaluable. Adverse events (AEs) and immune-related events of interest type and grade were reported according to the Common Terminology Criteria for Adverse Events (CTCAE) version 5.0: all the Aes between each cure were graded and notified in the report of the medical visit. The treatment was continued until disease progression according to the RECIST criteria, development of an unacceptable toxicity, lost to follow-up, withdrawal of consent or death. All patients were treated according to their own physician decisions.

### 2.3. Data Collection

We collected the following data from patients medical files: demographic data as gender, age, smoking status, medical history, tumor characteristics as primitive location, histological type, presence of histological variants, stage of disease, surgery status, data and type for each regimen as treatment administered before and after pembrolizumab, pembrolizumab rank of line, Eastern Cooperative Oncology Group (ECOG) performance status (PS) of patients at diagnosis and at the first cycle of pembrolizumab (cycle 1, C1), Bellmunt risk factors (ECOG PS score above 0, hemoglobin rate (Hb) of less than 10 g per deciliter (g/dL), presence of liver metastases and time since the completion or discontinuation of previous therapy of less than 3 months) [13,14], metastatic sites, blood test results at baseline, date of first and last cycles, date of radiological examinations, date and type of the best achieved response, type and grade of Aes, reason for treatment discontinuation, survival status.

### 2.4. End Points

The co-primary endpoints were OS and progression-free survival (PFS) at 6 months which were assessed in the whole population. OS was defined as the time from treatment beginning to death from any cause or last follow-up date. PFS was defined as the time from treatment start to disease progression (PD) or death from any cause.

Secondary endpoints were ORR, DOR, disease control rate (DCR) and safety. ORR was defined as the rate of patients who achieved a confirmed complete response (CR) or partial response (PR) as best response based on tumor imaging assessments during the treatment. The DOR was defined as the time from the best ORR to PD or death. DCR was defined as the percentage of patients who had a non-progressive disease at the end of follow-up. The safety of the treatment was assessed by the Aes reports and the grade of severity of the Aes during all the duration of the treatment.

### 2.5. Statistical Analysis

Continuous data are presented median (interquartile range [IQR]). Categorical data are presented as number (%). OS, PFS, and DOR were estimated with the use of the Kaplan–Meier method. In the analysis of OS, patients who were alive or lost to follow-up had their data censored at the time of last contact. In the analysis of PFS, patients who were alive and without progression disease or who were lost to follow-up had their data censored at the time of last tumor assessment. Cox univariate regression was used to evaluate the association between clinical and biological factors and outcomes. Cox multivariable models were built to assess the association between parameters and the outcomes. Parameters associated with OS and PFS on univariate analyses (at a significance of *p* < 0.20) were selected for multivariate analyses. For all analyses, *p* < 0.05 was considered statistically significant. Statistical analyses involved using SAS v9.4 (SAS Institute, Cary, NC, USA).

## 3. Results

### 3.1. Population and Treatment

Between 2018 and 2021, a total of 93 patients were screened. Among them, 15 patients were excluded from the study, due to ineligibility criteria: the final analysis was conducted on 78 patients. Patients’ characteristics are shown in Supplementary Table S1. Median age was 73 years old; 51 patients (65.4%) were current or former smokers. Seven patients (9%) had no risk factors, 24 (30.8%) had one risk factor, 29 (37.2%) had two risk factors and 18 (23.1%) had three or more risk factors; 43 patients (55.1%) had completed or discontinued the most recent therapy less than 3 months before starting of pembrolizumab. Bladder was the primitive cancer location for 62 patients (79.5%) and upper tract for 15 (19.2%). The most frequent pathological diagnosis was pure urothelial carcinoma (74 patients, 94.9%). Three patients (3.8%) had a sarcomatoid variant and one patient (1.3%) had a plasmacytoid variant. Fifteen patients (19%) received pembrolizumab as first line for metastatic disease after peri- or post-operative chemotherapy, 42 patients (54%) had pembrolizumab as second line, 12 patients (15%) as third line and 9 (12%) as fourth or subsequent line.

### 3.2. Overall

The median number of cycles of pembrolizumab received until the time of data cut-off was six cycles. At the time of data cut-off, nine patients were still receiving pembrolizumab. The median duration of therapy among the 69 patients who discontinued the treatment was 3.2 months (1.4–7.0), the mean duration of therapy was 5.1 months.

### 3.3. Overall Survival

**Figure 1:**
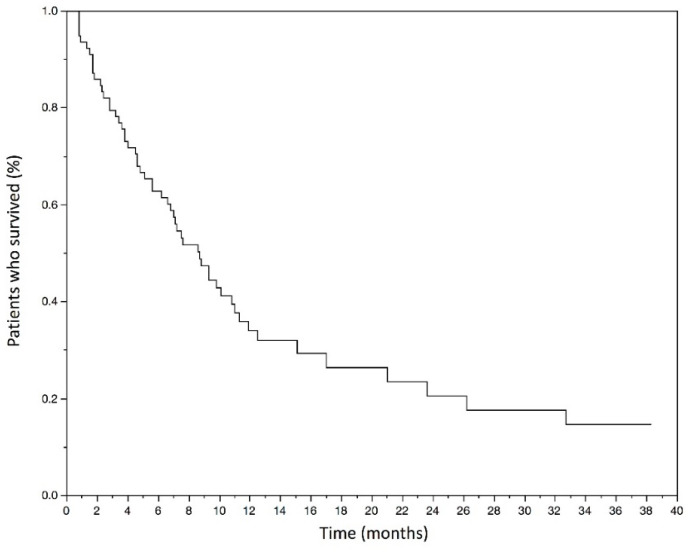
Kaplan–Meyer estimates of overall survival in months in the studied population.

At the time of data cut-off, 55 deaths (70.5%) occurred in the study population. The mOS was 7.3 months (3.8–12.2) and the mean OS was 10 months. The Kaplan–Meyer graphic estimated OS rate at 6 months was 61.5% (50.5–72.6) (Figure 1).

The median overall survival was 7.3 months (3.8–12.2). The estimated overall survival rate at 6 months was 61.5% (50.5–72.6).

Statistically significant variables (*p* < 0.20) at univariate analysis (Supplementary Table S2), which have been included in multivariate analysis were: ECOG PS score at diagnosis and at C1, type of first line of treatment, previous BCG therapy, time since most recent chemotherapy before pembrolizumab, presence of lung, liver and bone metastases before starting of pembrolizumab, Hb level at C1 and number of Bellmunt risk factors at C1.

At the multivariate analysis for death (Table 1), patients with ECOG PS score 0 or 1 at diagnosis had a better OS than patients with ECOG PS score of 2 (hazard ratio [HR] 5.78, 95% CI 1.02–12.65, *p* = 0.047). The levels of Hb at cycle 1 were associated with a better OS (HR 0.57 per unit, 95% CI 0.33–0.91, *p* = 0.027). However, the presence of liver and bone metastases at C1 was associated with a poorer OS (HR 9.46, 95% CI, 2.08–23.07, *p* = 0.004 and HR 2.52, 95% CI, 1.06–5.97, *p* = 0.036, respectively). Moreover, a number of Bellmunt risk factors ≥ 2 (HR 1.29, 95% CI 1.04–1.98, *p* = 0.007 for two risk factors and HR 2.04, 95% CI 1.05–3.61, *p* = 0.004 for three or more risk factors) and a time since most recent chemotherapy < 3 months were also associated with a poorer OS. There was no significant difference in survival according to the type of first line treatment, previous BCG therapy, presence of lung metastases at C1 and ECOG PS score at C1.

### 3.4. Progression-Free Survival

**Figure 2:**
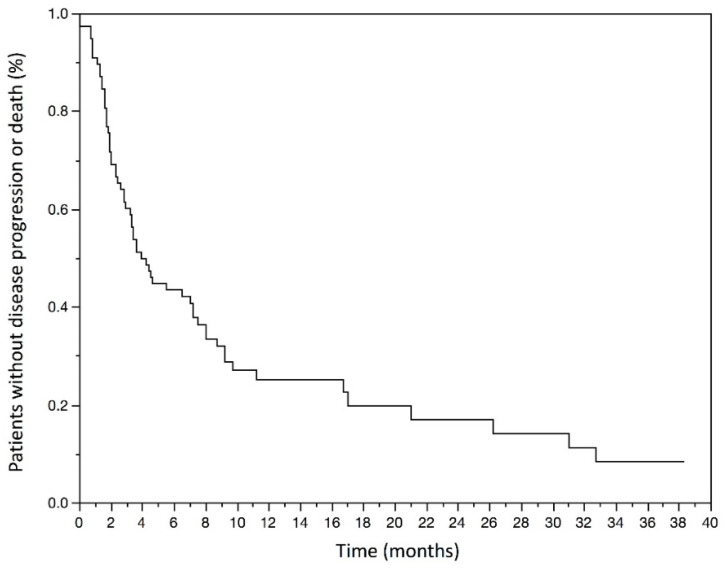
Kaplan–Meyer estimates of Progression-free survival in months in the studied population.

The mPFS was 3.1 (1.4–7.2) months and the mean PFS was 5.6 months in the population. The Kaplan–Meyer graphic estimated PFS rate at 6 months was 42.3% (31.1–53.5) (Figure 2).

The median progression-free survival was 3.1 (1.4–7.2) months. The estimated progression-free survival rate at 6 months was 42.3% (31.1–53.5).

Statistically significant variables (*p* < 0.20) associated with a shorter PFS at univariate analysis (Supplementary Table S3), which have been included in multivariate analysis were: age < 65 years old, previous BCG therapy, time since most recent chemotherapy before pembrolizumab, ECOG PS score at C1, presence of lung, liver and bone metastases at C1, number of Bellmunt risk factors at C1 and levels of Hb at C1.

At the multivariate analysis for death (Table 2), Hb level at C1 (HR 0.70, 95% CI 0.48–0.98, *p* = 0.049), time since most recent chemotherapy < 3 months (HR 3.85, 95% CI 1.06–14.03, *p* = 0.041), presence of bone metastases before pembrolizumab (HR 2.54, 95% CI 1.24–5.19, *p* = 0.011), were significantly associated with a poorer PFS. More often, a number of Bellmunt risk factors ≥ 2 (HR 1.33, 95% CI 1.02–3.24, *p* = 0.033 for two risk factors and HR 3.34, 95% CI 1.32–4.25, *p* = 0.028 for three or more risk factors) was also associated with a poorer PFS.

There was no significant difference in PFS according to age, previous BCG therapy, ECOG PS score at C1, and presence of liver or lung metastases.

### 3.5. Objective Response

In the whole study population, the best ORR was 35.9% (95% CI 25.0–46.8): 11 patients had a CR and 17 patients had a PR; moreover eight patients had a SD and 37 had a PD (47.44%) as best response.

The median time of best response was 2.0 [1.6–3.3] months (mean of 2.7 months). The median DOR was 17 days (0–97) and the mean DOR was 95.5 days (193.8). The DCR was 30.8% (24/78).

Atypical patterns of responses occurred during treatment with pembrolizumab, such as hyperprogression in 11 patients (14.10%) which led to a fast deterioration of the general state and discontinuation of the treatment for all cases, and one case of pseudoprogression (1.28%) which showed signs of progression at the tumor imaging assessment with an improvement of the general state. The patient who experienced a pseudoprogression was still treated with pembrolizumab at the time of data cut-off.

At the time of data cut-off, 50 patients discontinued pembrolizumab treatment because of PD, 9 because of toxicity, 3 because of CR and 7 for other reasons. Among patients who discontinued because of PD, the progression occurred after a median time of 2.7 [1.7–5.7] months (mean of 4.6 months) after beginning of treatment. Overall, the preferential sites of progression were lymph-nodes (76.0%), lung (48.0%), bone (48.0%), liver (46.0%), brain (6.0%) and other visceral localizations (4.0%).

Among the three patients with sarcomatoid variant, two of them were still receiving pembrolizumab at the time of data cut-off and had a CR and SD as best response, respectively; the third patient had a PD as best response. The patient with plasmocytoid variant discontinued treatment because of toxicity.

As to the first subsequent treatment after pembrolizumab failure, 25 patients switched treatment and among them: 15 patients switched to a taxane-based chemotherapy, three to a platinum-based chemotherapy, three patients were included into clinical trials, two switched to vinflunine and one to a fluorouracil-based chemotherapy. Among these patients, six of them continued treatment with a subsequent line.

### 3.6. Adverse Events

All AEs and events of interest attributed to pembrolizumab by the medical team are shown in Table 3.

AEs that were considered to be related to treatment occurred in 80.8% of the population. The most common treatment-related AEs of any grade were fatigue (46.2% of patients), diarrhea (11.5%), pruritus (10.3%) and nausea (9.0%). There were no grade 3 AEs that occurred with an incidence of 5% or more in the population. The most common events of interest reported were five cases of thyroid disorders, three cases of thyroiditis, one case of pancolitis, one case of pneumonitis and one case of adrenal insufficiency. Nine patients discontinued the treatment due to toxicity. No grade 4 or 5 AEs occurred during treatment with pembrolizumab.

## 4. Discussion

In our retrospective, real-life study, involving patients with advanced urothelial cancer that progressed during or after platinum-based chemotherapy, pembrolizumab showed a mOS of 7.3 months and an estimated OS rate at 6 months of 61.5%; moreover, the mPFS was 3.1 months with an estimated PFS rate at 6 months of 42.3%. Treatment with pembrolizumab was also associated with an ORR of 35.9% and an acceptable safety profile.

The results of our study are consistent with those of Keynote-045 trial [11], where OS rate at 6 months was 62.6% and PFS rate at 6 months was 27%. The mPFS in our study (3.1 months) is comparable to the mPFS found in the Keynote-045 trial (2.1 months). The median time of response in our study is comparable to the median time of response of patients included in Keynote-045 trial (2.0 vs. 2.1 months, respectively). We reported an ORR of 35.9%, higher than in Keynote-045 trial (21.9%).

The results of our study are also comparable to those of a Japanese study by Fujiwara and colleagues [15], confirming the effectiveness and good safety profile of pembrolizumab in advanced UC.

However, despite their utility, comparisons among trials should be interpreted with caution because of possible selection or confounding biases: differences in study population, methodology, presence of a control arm and randomization.

In our study, multivariate analysis showed that several factors could have an interesting prognostic value for OS and PFS. Presence of liver and bone metastases, ECOG PS score at diagnosis, Hb level and time since most recent chemotherapy seem to be important variables to be taken into consideration before treatment initiation. These variables confirm the relevance of using Bellmunt risk factors which identified liver metastases, Hb level < 10 g/dL, ECOG PS score > 0 and time since the discontinuation of previous chemotherapy < 3 months as prognostic factors [13]. In contrast to our findings, the Keynote-052 trial, that aimed to evaluate the safety and antitumor activity of first-line pembrolizumab in subgroups of cisplatin-ineligible older patients (aged ≥ 65 and ≥75 years old) with advanced UC, including those with ECOG PS 2, found that neither age nor poor PS appeared to have an impact on the efficacy of pembrolizumab [16]. Another study which assessed different PD-1/PD-L1 inhibitors in UC cisplatin-ineligible patients concluded that anemia and liver metastases were associated with a worse survival [17]. In our population, age and ECOG PS score at C1 were not found to be significant associated with prognosis, but ECOG PS score of 2 at diagnosis has been statistically associated with a poorer OS. Considering the number of patients in our study, a low statistical power could explain the difference of results compared to the literature.

Even if there is no consensual definition for hyperprogression, the rate of hyperprogression observed in our study that occurred during treatment with pembrolizumab was consistent with the literature (incidence varied between 4% and 29% of all responses) [18]. Hyperprogression could be defined as a strong accelerated tumor progression after initiation of immunotherapy, confirmed by a RECIST progression at the first evaluation, a time to treatment failure ≤ 2 months, and is associated to poor survival [19]. In contrast, pseudoprogression is defined as an increase in the size of the primary tumor or the appearance of a new lesion followed by tumor regression, in patients with an improved general status. Since pseudoprogression is diagnosed using retrospective imaging data, with the risk of premature cessation of immunotherapeutic treatment, the number of pseudoprogression cases may be underestimated, in both literature (incidence estimated at less than 10%) and in our study [20].

However, the continuation of pembrolizumab administration beyond progression might be beneficial in patients with metastatic UC who were clinically stable [21].

The incidence of AEs and events of interest with pembrolizumab was mostly of grade 1 or 2. Pembrolizumab appears to be well tolerated with a good safety profile. Grade 3 were relatively infrequent and few events resulted in the discontinuation of treatment. No death occurred because of toxicity related to pembrolizumab. All AEs reported were well-known and described in literature and pembrolizumab summary of product characteristics. Overall, there was no new or unexpected toxic effects with pembrolizumab [11,22,23].

To the best of our knowledge, this is the first European study to assess pembrolizumab treatment in advanced urothelial cancer in a real-world and larger population. Although RCTs are the gold standard for obtaining evidence of treatment’s efficacy and safety, real-life studies allow an assessment under normal conditions in daily clinical practice, which can provide complementary evidence in a heterogeneous population of patients. Moreover, our cohort represents the largest single-center analysis of UC in the country.

Despite the important advances that have been made in the last years, many gaps still exist in treatment decision strategies of advanced UC: among them, patients’ selection criteria, predictive and prognostic factors of response to immunotherapy, histological variants’ sensibility to immunotherapy and treatment duration. Our study aimed to give an answer to some of these questions or an orientation on how to deal with every-day UC patients. However multicenter studies enrolling more patients should be performed in order to better clarify some points.

### Limitations Section

Several limitations of our study warrant mention. First, we evaluated the clinical practice data related to the efficacy and tolerability of pembrolizumab after the failure of platinum-based chemotherapy for metastatic UC in a retrospective, non-randomized, trial. The design of the study increased the potential risk of bias and missing data. Therefore, there was a possible bias in extracting the prognostic factors. Thus, further large-scale investigations with greater statistical power are still required to confirm our observations and validate them in clinical practice. The lack of control group is a major limitation in our study. The efficacy and safety of the treatment could not be confirmed by comparing the results with a control group. The effectiveness of our findings should be confirmed by controlled randomized clinical trials. Second, data quality may not be comparable to data derived from randomized controlled trials; in particular, despite the use of RECIST criteria, imaging did not follow a strict time schedule as is the case in clinical trials; thus, a simple comparison between our results and those of clinical trials might be difficult. Third, a variety of inclusion and exclusion criteria do not apply in this real-world setting, hence data are less homogenous. Moreover, the median observation period for the present study was short at 6 months; a longer observation period might be necessary.

## 5. Conclusions

In conclusion, our results seem to confirm the previous RCTs in the literature, with a high rate of objective response, 6-months OS and PFS, and a good safety profile of pembrolizumab in patients with advanced UC that progressed after platinum-based chemotherapy.

Overall, pembrolizumab is a promising therapeutic line, used in daily clinical practice in our center, and included in several clinical trials to improve scientific knowledge and achieve a better therapeutic care for UC patients.

## Figures and Tables

**Table 1:** OS status at 6 months—Multivariate analysis.

Parameters	HR (95% CI)	*p* Value
**Sex**		
**Female**	Ref.	
**Male**	1.87 (0.74–4.76)	0.185
**ECOG PS score at diagnosis**		
**0**	Ref.	
**1**	2.87 (0.87–9.43)	0.081
**2**	5.78 (1.02–12.65)	**0.047**
**Previous BCG therapy**		
**No**	Ref.	
**Yes**	1.63 (0.62–4.30)	0.320
**Time since most recent chemotherapy**		
**≥3 months**	Ref.	
**<3 months**	7.15 (1.51–23.81)	**0.013**
**ECOG PS score at C1**		0.186
**0**	Ref.	
**1**	2.90 (0.47–17.68)	0.248
**2**	1.68 (0.22–12.52)	0.612
**Presence of metastases at C1**		
**Bone**	2.52 (1.06–5.97)	**0.036**
**Liver**	9.46 (2.08–23.07)	**0.004**
**Lung**	1.51 (0.59–3.80)	0.386
**Bellmunt risk factors at C1**		0.467
**0–1**	Ref.	
**2**	1.29 (1.04–1.98)	**0.007**
**≥3**	2.04 (1.05–3.61)	**0.004**
**Hb level at C1**	0.57 (0.33–0.91)	**0.027**

**Table 2:** PFS status at 6 months—Multivariate analysis.

Parameters	HR (95% CI)	*p* Value
**Age**		
**>65 years old**	Ref.	
**<65 years old**	1.88 (0.48–0.98)	0.078
**Previous BCG therapy**		
**No**	Ref.	
**Yes**	1.64 (0.77–3.49)	0.197
**ECOG PS score at C1**		0.176
**0**	Ref.	
**1**	2.73 (0.75–10.03)	0.128
**2**	4.06 (0.93–17.78)	0.063
**Time since most recent chemotherapy**		
**≥3 months**	Ref	
**<3 months**	3.85 (1.06–14.03)	**0.041**
**Presence of metastases at C1**		
**Bone**	2.54 (1.24–5.19)	**0.011**
**Liver**	2.32 (0.74–7.24)	0.149
**Lung**	1.70 (0.84–3.43)	0.141
**Bellmunt risk factors at C1**		0.858
**0–1**	Ref.	
**2**	1.33 (1.02–3.24)	**0.033**
**≥3**	3.34 (1.32–4.25)	**0.028**
**Hb at C1**	0.70 (0.48–0.98)	**0.049**

**Table 3:** Adverse Events in the whole population treated with pembrolizumab.

	Any Grade		Grade 3	
	N	%	N	%
**AEs**				
Any event	63	80.8	7	9.0
**Grade of AEs**				
1	41	52.6	0	0.0
2	15	20.5	0	0.0
3	7	9.0	7	9.0
**Type of AEs**				
Fatigue	36	46.2	4	5.1
Diarrhea	9	11.5	2	2.6
Pruritus	8	10.3	0	0.0
Nausea	7	9.0	1	1.3
Constipation	6	7.7	1	1.3
Weight loss	6	7.7	1	1.3
Anorexia	6	7.7	0	0.0
Peripherical sensory neuropathy	5	6.4	1	1.3
Edema limbs	5	6.4	0	0.0
Vomiting	4	5.1	1	1.3
Skin and subcutaneous tissue disorders (rash maculo-papular, skin ulceration, dry skin…)	4	5.1	1	1.3
Hypercalcemia	4	5.1	0	0.0
Acute kidney injury	3	3.9	2	2.6
Hepatobiliary disorders	3	3.9	1	1.3
Platelet count decreased	2	2.6	0	0.0
Neutrophil count decreased	2	2.6	0	0.0
Anemia	1	1.3	0	0.0
Cough	1	1.3	0	0.0
**Event of interest**				
Hyperthyroidism	3	3.9	0	0.0
Thyroïditis	3	3.9	0	0.0
Hypothyroidism	2	2.6	0	0.0
Pancolitis	1	1.3	1	1.3
Pneumonitis	1	1.3	0	0.0
Adrenal insufficiency	0	0.0	1	1.3

The AEs are listed in descending order of frequency. The events of interest are AEs with an immune-related cause. They are listed in descending order of frequency.

## Data Availability

The data presented in this study are available on request from the corresponding author.

## Supplementary Materials

The following supporting information can be downloaded at: https://www.mdpi.com/article/10.3390/curroncol29020080/s1, Table S1: Baseline characteristics of the study population; Table S2: OS status at 6 months: univariate analysis; Table S3: PFS status at 6 months: univariate analysis.
