# Supplementary material for: Clinical Benefit of Pembrolizumab in Advanced Urothelial Cancer Patients in Real-Life Setting: An Efficacy and Safety Monocentric Study
Source: Curr Oncol. 2022 Feb 10;29(2):945–55. doi: 10.3390/curroncol29020080 (PMC8870973; doi:10.3390/curroncol29020080)
Supplement: Supplementary file 1 [file curroncol-29-00080-s001.zip › curroncol-1573188-supplementary.pdf]

Supplementary Table S1. Baseline characteristics of the study population.

Characteristic	*No. (%)*
**Age – Year**	
Median	73 [42 - 89]
**Sex**	
Female	21 (26.9)
Male	57 (73.1)
**ECOG PS score at diagnosis**	
0	27 (34.6)
1	44 (56.4)
2	7 (9.0)
**Smoking history**	
Yes	51 (65.4)
No	27 (34.6)
**No. of Bellmunt risk factors**	
0	7 (9.0)
1	24 (30.8)
2	29 (37.2)
≥3	18 (23.0)
**Completion or discontinuation of most recent therapy <3 months**	43 (55.1)
**Primitive location**	
Bladder	62 (79.5)
Upper tract	15 (19.2)
Urethra	1 (1.3)
**Histology**	
Pure urothelial	72 (92.3)
Other histology variant	6 (7.7)
**Stage at diagnosis**	
Non-muscle invasive UC	23 (29.5)
Muscle invasive UC	48 (61.5)
Metastatic UC	7 (9.0)
**Previous BCG therapy**	
Yes	21 (25.9)
No	57 (74.1)
**Peri-operative chemotherapy**	
Yes	20 (25.6)
No	58 (74.4)
**Type of peri-operative chemotherapy**	
MVAC	8 (40)
Cisplatin-Gemcitabine	7 (35)
Carboplatin-Gemcitabine	4 (20)
Paclitaxel-Gemcitabine	1 (5)
**Post-operative chemotherapy**	
Yes	13 (16.7%)
No	65 (83.3)
**Type of post-operative chemotherapy**	
MVAC	7 (54)
Paclitaxel-Gemcitabine	4 (31)
Carboplatin-Gemcitabine	2 (15)
**Line of pembrolizumab as metastatic treatment**	
1	15 (19)
2	42 (54)

3	12 (15)
≥4	9 (12)
**EGOG PS score at C1**	
0	15 (19.2)
1	51 (65.4)
2	12 (15.4)
Abbreviations: BCG: Bacillus Calmette-Guerin; MVAC: methotrexate, vinblastine, doxorubicin and cisplatin.	

Supplementary Table S2. OS status at 6 months: univariate analysis.

	Dead	N=30	Alive	N=48		
	N	%	N	%	HR (CI 95%)	P value
**Sex**						
Female	6	20.00%	15	31.25%	Ref.	
Male	24	80.00%	33	68.75%	1.71 [0.70 – 4.21]	0.235
**Age 65**						
≥65 years old	22	73.33%	36	75.00%	Ref.	
<65 years old	8	26.67%	12	25.00%	1.05 [0.47 – 2.36]	0.907
**Smoking history**						0.867
No	8	26.67%	10	20.83%	Ref.	
Yes	19	63.33%	32	66.67%	0.85 [0.37 - 1.95]	0.703
Unknown	3	10.00%	6	12.50%	0.71 [0.19 – 2.68]	0.616
**Cardiovascular disorders***						
No	10	33.33%	20	41.67%	Ref.	
Yes	20	66.67%	28	58.33%	1.25 [0.58 – 2.67]	0.569
**Former or current cancer other than urinary**						
No	23	76.67%	41	85.42%	Ref.	
Yes	7	23.33%	7	14.58%	1.54 [0.31 – 3.60]	0.314
**Metabolic disorders****						
No	21	70.00%	33	68.75%	Ref.	
Yes	9	30.00%	15	31.25%	0.87 [0.40 – 1.89]	0.724
**Renal impairment**						
No	29	96.67%	45	93.75%	Ref.	
Yes	1	3.33%	3	6.25%	0.58 [0.08 – 4.27]	0.594
**Primitive location**						0.865
Bladder	25	83.33%	37	77.08%	Ref.	
Upper tract	5	16.67%	10	20.83%	0.77 [0.29 – 2.00]	0.590
Urethra	0	0.00%	1	2.08%	-	
**Stage at diagnosis**						0.738
Non-muscle invasive UC	10	33.33%	13	27.08%	Ref.	
Muscle invasive UC	17	56.67%	31	64.68%	0.76 [0.35 – 1.66]	0.487
Metastatic UC	3	10.00%	4	8.33%	1.05 [0.30 – 3.82]	0.939
**Histology**						
Pure urothelial	28	93.33%	44	91.67%	Ref.	
Other	2	6.67%	4	8.33%	0.72 [0.17 – 3.03]	0.657
**Variant**						
No	29	96.67%	45	93.75%	Ref.	
Yes	1	3.33%	3	6.25%	0.53 [0.07 – 3.92]	0.538
**ECOG PS score at diagnosis**						0.071
0	6	20.00%	21	43.75%	Ref.	
1	20	66.67%	24	50.00%	2.49 [0.99 – 6.21]	0.050
2	4	13.33%	3	6.25%	3.87 [1.09 – 13.72]	0.037
**Previous BCG therapy**						
No	18	60.00%	39	81.25%	Ref.	
Yes	12	40.00%	9	18.75%	2.17 [1.04 – 4.51]	0.038
**Perioperative chemotherapy**						
No	25	83.33%	34	70.83%	Ref.	
Yes	5	16.67%	14	29.17%	0.58 [0.22 – 1.51]	0.265

**Schema of perioperative chemotherapy**						NA
MVAC	4	80.00%	4	26.67%		
Cisplatin-Gemcitabine	0	0.00%	7	46.67%		
Carboplatin-Gemcitabine	1	20.00%	3	20.00%		
Paclitaxel-Gemcitabine	0	0.00%	1	6.67%		
**Surgery of primitive tumor**						
No	11	36.67%	12	25.00%	Ref.	
Yes	19	63.33%	36	75.00%	0.61 [0.29 – 1.30]	0.204
**Postoperative chemotherapy**						
No	26	86.67%	39	81.25%	Ref.	
Yes	4	13.33%	9	18.75%	0.62 [0.22 – 1.79]	0.382
**Schema of postoperative chemotherapy**						NA
MVAC	2	50.00%	5	55.56%		
Carboplatin-Gemcitabine	1	25.00%	1	11.11%		
Paclitaxel-Gemcitabine	1	25.00%	3	33.33%		
**Type of first-line metastatic treatment**						
Chemotherapy	26	86.67%	35	72.92%	Ref.	
Immunotherapy	4	13.33%	13	27.08%	0.49 [0.17 – 1.41]	0.185
**First-line metastatic treatment**						NA
MVAC	6	22.22%	12	31.58%		
Cisplatin-Gemcitabine	6	22.22%	6	15.79%		
Carboplatin-Gemcitabine	9	33.33%	10	26.32%		
Paclitaxel-Gemcitabine	4	14.81%	7	18.42%		
Carboplatin-Paclitaxel	1	3.70%	0	0.00%		
**Time since most recent chemotherapy**						
≥3 months	10	33.33%	25	52.08%	Ref.	
<3 months	20	66.67%	23	47.92%	1.85 [0.86 – 3.95]	0.113
**ECOG PS score at C1**						0.071
0	3	10.00%	12	25.00%	Ref.	
1	20	66.67%	31	64.58%	2.12 [0.63 – 7.15]	0.224
2	7	23.33%	5	10.42%	4.49 [1.16 – 17.40]	0.029
**Site of metastasis at C1: Lymph node**						
No	4	13.33%	3	6.25%	Ref.	
Yes	26	86.67%	45	93.75%	0.55 [0.19 – 1.57]	0.261
**Site of metastasis at C1: Lung**						
No	17	56.67%	34	70.83%	Ref.	
Yes	13	43.33%	14	29.17%	1.74 [0.84 – 3.59]	0.133
**Site of metastasis at C1:Liver**						
No	21	70.00%	43	89.58%	Ref.	
Yes	9	30.00%	5	10.42%	3.15 [1.43 – 6.91]	0.004
**Site of metastasis at C1: Bone**						
No	14	46.67%	39	81.25%	Ref.	
Yes	16	53.33%	9	18.75%	3.31 [1.61 – 6.80]	0.001
**Site of metastasis at C1: Brain**						nA
No	30	100.00%	47	97.92%		
Yes	0	0.00%	1	2.08%		
**Hb at C1**	10.5	[9.6-11.3]	11.5	[10-13]	0.79 [0.62 – 0.97]	0.025

**Hb<10**						
No	20	66.67%	36	75.00%	Ref.	
Yes	10	33.33%	12	25.00%	1.41 [0.66 – 3.01]	0.376
**ECOG PS score at C1 (>0)**						
No	4	13.33%	12	25.00%	Ref.	
Yes	26	86.67%	36	75.00%	1.79 [0.62 – 5.14]	0.278
**Bellmunt risk factors**						0.123
0 or 1	9	30.00%	22	45.83%	Ref.	
2	11	36.67%	18	37.50%	1.40 [0.58 – 3.37]	0.457
≥3	10	33.33%	8	16.67%	2.51 [1.02 – 6.20]	0.045
**Pembrolizumab administered as:**						0.352
1st line	3	10.00%	12	25.00%	Ref.	
2nd line	18	60.00%	24	50.00%	2.36 [0.69 – 8.02]	0.169
≥3rd line	9	30.00%	12	25.00%	2.49 [0.67 – 9.22]	0.171

* Cardiovascular disorders include heart failure, deep vein thrombosis, pulmonary embolism, coronary artery disease, arrhythmias, high blood pressure.

** Metabolism disorders include common endocrinology diseases such as diabetes, hypercholesterolemia, familial dyslipidemia, thyroid disorders, adrenal insufficiency.

Abbreviations: UC: urothelial carcinoma; ECOG PS: Eastern Cooperative Oncology Group Performance Status; BCG: Bacillus Calmette-Guerin; MVAC: methotrexate, vinblastine, doxorubicin and cisplatin.

Supplementary Table S3. PFS status at 6 months: univariate analysis.

	Dead	N=30	Alive	N=48		
	N	%	N	%	HR (IC 95%)	P value
**Sex**						
Female	10	22.22%	11	33.33%	Ref.	
Male	35	77.78%	22	66.67%	1.42 [0.70 – 2.88]	0.327
**Age 65**						
≥65 years old	31	68.89%	27	81.82%	Ref.	
<65 years old	14	31.11%	6	18.18%	1.55 [0.81 - 2.92]	0.179
**Smoking history**						
No	11	24.44%	7	21.21%	Ref.	
Yes	28	62.22%	23	69.70%	0.79 [0.39 – 1.58]	0.502
Unknown	6	13.33%	3	9.09%	0.68 [0.24 – 1.97]	0.484
**Cardiovascular disorders**						
No	17	37.78%	13	39.39%	Ref.	
Yes	28	62.22%	20	60.61%	1.18 [0.64 – 2.18]	0.596
**Former or current cancer other than urinary**						
No	36	80.00%	28	84.85%	Ref.	
Yes	9	20.00%	5	15.15%	1.25 [0.60 – 2.59]	0.555
**Metabolic disorders**						
No	32	71.11%	22	66.67%	Ref.	
Yes	13	28.89%	11	33.33%	0.76 [0.39 – 1.47]	0.415
**Renal impairment**						
No	43	95.56%	31	93.94%	Ref.	
Yes	2	4.44%	2	6.06%	0.74 [0.18 – 3.07]	0.682
**Primitive location**						0.868
Bladder	35	77.78%	27	81.82%	Ref.	
Upper tract	9	20.00%	6	18.18%	1.07 [0.51 - 2.23]	0.859
Urethra	1	2.22%	0	0.00%	1.69 [0.23 – 12.40]	0.607
**Stage at diagnosis**						0.303
Non-muscle invasive UC	16	35.56%	7	21.21%	Ref.	
Muscle invasive UC	26	57.78%	22	66.67%	0.62 [0.33 – 1.17]	0.139
Metastatic UC	3	6.67%	4	12.12%	0.57 [0.17 – 1.96]	0.371
**Histology**						
Pure urothelial	43	95.56%	29	87.88%	Ref.	
Other	2	4.44%	4	12.12%	0.39 [0.10 – 1.64]	0.202
**Variant**						
No	44	97.78%	30	90.91%	Ref.	
Yes	1	2.22%	3	9.09%	0.29 [0.04 – 2.12]	0.224
**ECOG PS score at diagnosis**						0.407
0	16	35.56%	11	33.33%	Ref.	
1	24	53.33%	20	60.61%	1.10 [0.58 – 2.09]	0.773
2	5	11.11%	2	6.06%	1.98 [0.72 – 5.44]	0.188
**Previous BCG therapy**						
No	28	62.22%	29	87.88%	Ref.	
Yes	17	37.78%	4	12.12%	2.38 [1.29 – 4.40]	0.006
**Perioperative chemotherapy**						
No	37	82.22%	22	66.67%	Ref.	
Yes	8	17.78%	11	33.33%	0.66 [0.31 – 1.43]	0.294
**Schema of perioperative chemotherapy**						NA

MVAC	6	75.00%	2	16.67%		
Cisplatin-Gemcitabine	1	12.50%	6	50.00%		
Carboplatin-Gemcitabine	1	12.50%	3	25.00%		
Paclitaxel-Gemcitabine	0	0.00%	1	8.33%		
**Surgery of primitive tumor**						
No	15	33.33%	8	24.24%	Ref.	
Yes	30	66.67%	25	75.76%	0.67 [0.36 – 1.26]	0.218
**Postoperative chemotherapy**						
No	37	82.22%	28	84.85%	Ref.	
Yes	8	17.78%	5	15.15%	0.83 [0.37 - 1.87]	0.659
**Schema of postoperative chemotherapy**						0.943
MVAC	5	62.50%	2	40.00%	Ref.	
Carboplatin-Gemcitabine	1	12.50%	1	20.00%	0.75 [0.08 – 6.76]	0.798
Paclitaxel-Gemcitabine	2	25.00%	2	40.00%	0.78 [0.14 – 4.27]	0.774
**Type of first-line metastatic treatment**						
Chemotherapy	36	80.00%	25	75.76%	Ref.	
Immunotherapy	9	20.00%	8	24.24%	0.78 [0.36 – 1.67]	0.523
**First-line metastatic treatment**						NA
MVAC	8	21.05%	10	37.04%		
Cisplatin-Gemcitabine	8	21.05%	4	14.81%		
Carboplatin-Gemcitabine	13	34.21%	6	22.22%		
Paclitaxel-Gemcitabine	6	15.79%	5	18.52%		
Carboplatin-Paclitaxel	1	2.63%	0	0.00%		
**Time since most recent chemotherapy**						
≥3 months	18	40.00%	17	51.52%	Ref.	
<3 months	27	60.00%	16	48.48%	1.65 [0.90 – 3.04]	0.105
**ECOG PS score at C1**						0.023
0	5	11.11%	10	30.30%	Ref.	
1	31	68.89%	20	60.61%	1.94 [0.75 – 5.00]	0.169
2	9	20.00%	3	9.09%	4.30 [1.44 – 12.90]	0.009
**Site of metastasis at C1: Lymph node**						
No	4	8.89%	3	9.09%	Ref.	
Yes	41	91.11%	30	90.91%	0.81 [0.29 – 2.26]	0.683
**Site of metastasis at C1: Lung**						
No	27	60.00%	24	72.73%	Ref.	
Yes	18	40.00%	9	27.27%	1.64 [0.90 – 2.98]	0.109
**Site of metastasis at C1: Liver**						
No	35	77.78%	29	87.88%	Ref.	
Yes	10	22.22%	4	12.12%	1.95 [0.96 - 3.95]	0.064
**Site of metastasis at C1: Bone**						
No	24	53.33%	29	87.88%	Ref.	
Yes	21	46.67%	4	12.12%	2.83 [1.55 – 5.16]	<0.001
**Site of metastasis at C1: Brain**						NA
No	45	100.00%	32	96.97%		
Yes	0	0.00%	1	3.03%		
**Hb at C1**	10.7	[9.7-12.1]	11.5	[9.8-12.9]	0.87 [0.73 – 1.03]	0.109
**Hb<10**						
No	32	71.11%	24	72.73%	Ref.	

Yes	13	28.89%	9	27.27%	1.14 [0.59 – 2.17]	0.701
**ECOG PS score at C1 (>0)**						
No	6	13.33%	10	30.30%	Ref.	
Yes	39	86.67%	23	69.70%	1.81 [0.76 – 4.28]	0.179
**Bellmunt risk factors**						0.174
0 or 1	17	37.78%	14	42.42%	Ref.	
2	15	33.33%	14	42.42%	1.14 [0.56 – 2.30]	0.723
≥ 3	13	28.89%	5	15.15%	1.96 [0.94 – 4.07]	0.073
**Pembrolizumab administered as:**						0.499
1st line	7	15.56%	8	24.24%	Ref.	
2nd line	26	57.78%	16	48.48%	1.69 [0.70 – 4.12]	0.245
≥3rd line	12	26.67%	9	27.27%	1.63 [0.61 - 4.35]	0.327

* Cardiovascular disorders include heart failure, deep vein thrombosis, pulmonary embolism, coronary artery disease, arrhythmias, high blood pressure.
** Metabolism disorders include common endocrinology diseases such as diabetes, hypercholesterolemia, familial dyslipidemia, thyroid disorders, adrenal insufficiency.
Abbreviations: UC: urothelial carcinoma; ECOG PS: Eastern Cooperative Oncology Group Performance Status; BCG: Bacillus Calmette-Guerin; MVAC: methotrexate, vinblastine, doxorubicin and cisplatin.
